# Structure of PatF from *Prochloron didemni*

**DOI:** 10.1107/S1744309113012931

**Published:** 2013-05-23

**Authors:** Andrew F. Bent, Jesko Koehnke, Wael E. Houssen, Margaret C. M. Smith, Marcel Jaspars, James H. Naismith

**Affiliations:** a Biomedical Sciences Research Complex, University of St Andrews, North Haugh, St Andrews, Fife KY16 9ST, Scotland; b Marine Biodiscovery Centre, Department of Chemistry, University of Aberdeen, Meston Walk, Aberdeen AB24 3UE, Scotland; c Institute of Medical Sciences, University of Aberdeen, Ashgrove Road West, Aberdeen AB25 2ZD, Scotland; d Department of Biology, University of York, Wentworth Way, York YO10 5DD, England

**Keywords:** patellamide, cyanobactins, natural products, prenyltransferases

## Abstract

Patellamides are macrocyclic peptides with potent biological effects and are a subset of the cyanobactins. Cyanobactins are natural products that are produced by a series of enzymatic transformations and a common modification is the addition of a prenyl group. Puzzlingly, the pathway for patellamides in *Prochloron didemni* contains a gene, *patF*, with homology to prenylases, but patellamides are not themselves prenylated. The structure of the protein PatF was cloned, expressed, purified and determined. Prenylase activity could not be demonstrated for the protein, and examination of the structure revealed changes in side-chain identity at the active site. It is suggested that these changes have inactivated the protein. Attempts to mutate these residues led to unfolded protein.

## Abstract 2

The X-ray crystal structure of PatF from *P. didemni* was solved by the single-wavelength anomalous diffraction method to a resolution of 2.13 Å.

## 1. Introduction

Medicinal chemists have for many decades looked towards natural products as a scaffold for the design of new drug molecules; however, their synthesis can often be challenging. The exploration of natural product biosynthetic pathways allows the potential exploitation of these pathways to produce new chemical entities. In order to achieve this, study of the individual enzymes involved can give great insight into both their function and the potential to manipulate them for novel purposes.

**Figure 1:**
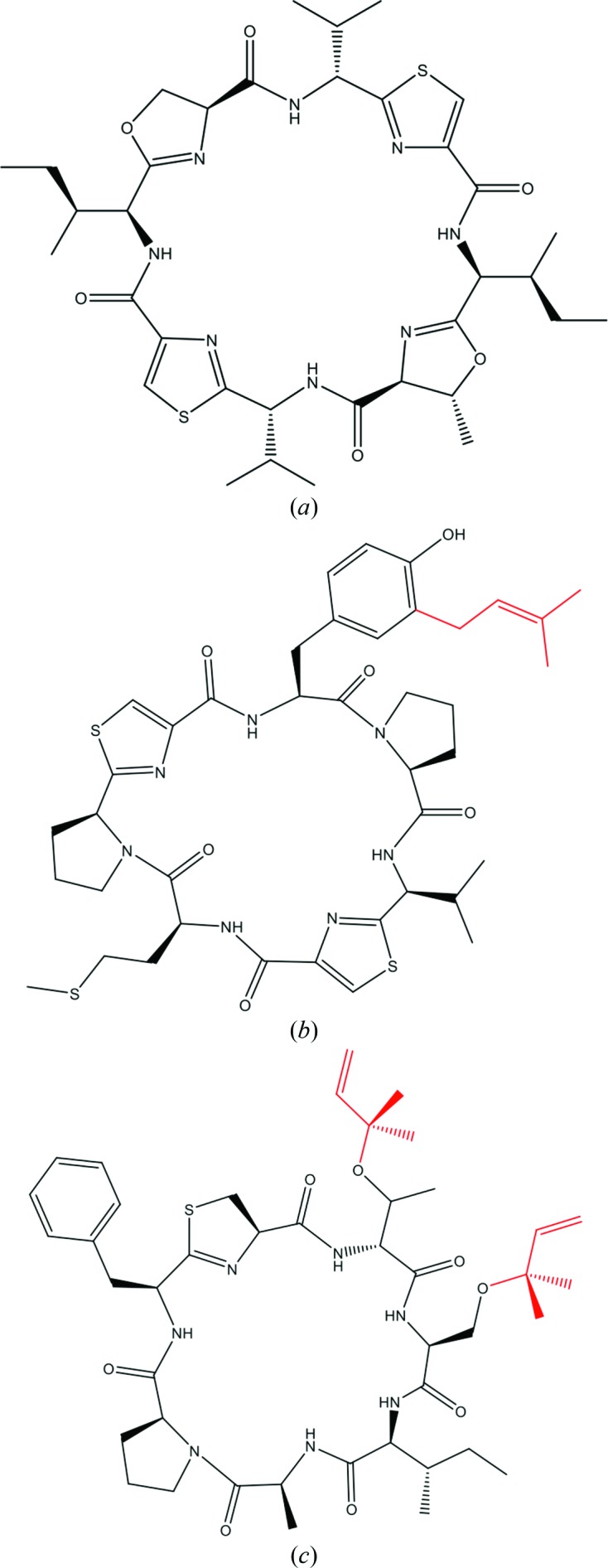
Structures of cyanobactins: (*a*) patellamide A, (*b*) an aestuaramide and (*c*) trunkamide A. Prenyl groups are coloured red.

The cyanobactins, which are a natural product superfamily, are cyclic peptides containing a range of post-translational modifications including heterocyclization, epimerization and prenylation, and have a wide range of biological activities (Sivonen *et al.*, 2010 ▶). The most widely studied members of the cyanobactins are the patellamides produced by *Prochloron didemni*, an obligate symbiont of the sea squirt *Lissoclinum patella*. The patellamides are eight-residue cyclic peptides containing oxazolines, thiazoles and d-amino acids and are derived from a ribosomally produced precursor peptide. This peptide is chemically modified by a series of enzymes that includes proteases, heterocyclases and macrocyclases (Fig. 1 ▶ *a*; Houssen & Jaspars, 2010 ▶). The patellamide gene cluster has been identified (seven genes; *patA*–*patG*) and the majority of the proteins or their contributing domains have been assigned specific functions (Schmidt *et al.*, 2005 ▶). The crystal structures of the protease domain of PatA and the macrocyclase domain of PatG have previously been reported and their mechanisms have been elucidated (Koehnke *et al.*, 2012 ▶; Houssen *et al.*, 2012 ▶; Agarwal *et al.*, 2012 ▶).

PatF has been shown to be essential for patellamide production *in vivo*; however, to date its function has not been confirmed (Donia *et al.*, 2006 ▶). Cyanobactin family members related to PatF have been shown to possess prenyltransferase activity. LynF from *Lyngbya aestuarii* prenylates tyrosine residues on the aestuaramide family of cyanobactins *via* a Claisen rearrangement (Fig. 1 ▶ *b*; McIntosh *et al.*, 2011 ▶; McIntosh *et al.*, 2013 ▶), while TruF1 from *Prochloron* spp. prenylates threonine and serine residues on the trunkamides (Fig. 1 ▶ *c*; Tianero *et al.*, 2012 ▶). These enzymes have sequence homologies to PatF of 44 and 41%, respectively.

Protein prenylation is a common post-translational modification that plays a role in protein–protein interactions and membrane tethering (Novelli & D’Apice, 2012 ▶). In cyanobactins, prenylation of hydroxyl groups occurs *via* a 3-methyl-but-2-en-1-yl group derived from dimethylallyl pyrophosphate (DMAPP) and is catalyzed by the cyanobactin-specific family of prenyltransferases (McIntosh *et al.*, 2011 ▶).

To date, no prenylation of isolated natural patellamides is evident. No tyrosine residues are found in natural patellamides and therefore if PatF is mechanistically similar to LynF then there is no substrate to prenylate. Secondly, threonines and serines are heterocyclized at an early stage in the patellamide-biosynthetic pathway, eliminating them as substrates. In the closely related trunkamide pathway these residues are not heterocyclized and are prenylated by TruF1.

Here, we report the crystal structure of PatF, the first from this cyanobactin enzyme family, to a resolution of 2.1 Å and provide evidence to suggest that it is likely to be an inactive prenyltransferase.

## 2. Methods

### 2.1. Expression and purification

The full-length *patF* gene from *P. didemni* was synthesized and cloned in pJexpress 411 plasmid (DNA 2.0) with an N-­terminal His_6_ tag and a *Tobacco etch virus* (TEV) protease site. The resulting protein was expressed in *Escherichia coli* BL21 (DE3) cells grown on auto-induction medium using the method of Studier (2005 ▶). Cultures were grown for 48 h at 293 K.

l-Selenomethionine-labelled (SeMet) PatF was expressed in *E. coli* BL21 (DE3) cells, cultures of which were grown in minimal medium supplemented with glucose-free nutrient mix (Molecular Dimensions) and 5% glycerol. The medium was inoculated with an overnight culture grown in Luria–Bertani (LB) medium and washed three times with minimal medium. After 15 min of growth at 310 K, 60 mg l^−1^ l-selenomethionine was added to the cultures. An amino-acid mix (100 mg l^−1^ lysine, phenylalanine and threonine, 50 mg l^−1^ isoleucine and valine) was added to the cultures when they reached an OD_600 nm_ of 0.6. After 15 min of further growth at 310 K the cultures were induced with 1 m*M* isopropyl β-d-1-thiogalacto­pyranoside (IPTG) and grown for 30 h at 293 K.

For native and SeMet PatF, cells were harvested by centrifugation (4000*g*, 277 K, 20 min) and resuspended in lysis buffer [150 m*M* NaCl, 20 m*M* Tris–HCl pH 8.0, 20 m*M* imidazole pH 8.0, 0.1%(*v*/*v*) Triton X-100, 3 m*M* β-mercaptoethanol (BME)] with the addition of Complete EDTA-free protease-inhibitor tablets (Roche) and 0.4 mg DNAse I (Sigma) per gram of wet cells. They were then lysed by passage through a cell disrupter at 207 MPa (Constant Systems). The lysate was cleared by centrifugation (40 000*g*, 277 K, 20 min) and loaded onto an Ni-Sepharose 6 FF column (GE Healthcare) equilibrated in lysis buffer. The column was washed with lysis buffer and PatF was eluted with 250 m*M* imidazole in the same buffer. The elution peak was passed over a desalting column pre-equilibrated in 150 m*M* NaCl, 10 m*M* HEPES pH 7.4, 1 m*M* TCEP, 10% glycerol. TEV protease was added at a mass ratio of 1:5 and the protein was digested for 2 h at 293 K to remove the His_6_ tag. The cleaved protein was loaded onto a second nickel column and PatF was found in the flowthrough. The protein was concentrated (Vivaspin concentrators, 10K molecular-weight cutoff) and applied onto a Superdex 75 gel-filtration column (GE Healthcare) equilibrated in the desalting column buffer. The protein eluted as a monomer and was confirmed by SDS–PAGE and mass spectrometry (MS). SeMet PatF was additionally confirmed to contain the expected three selenomethionine residues by mass spectrometry. The protein was of full length with the exception of the N-terminal methionine, which was removed during TEV protease cleavage, and the addition of an Arg and a Ser residue at the C-­terminus, which were artifacts from cloning.

### 2.2. Crystallization, structure solution and refinement

Crystals of SeMet PatF were grown in a condition consisting of 0.1 *M* sodium/potassium tartrate, 26% PEG 2K MME at 293 K using the hanging-drop vapour-diffusion method. The quality of the crystals was found to be improved following iterative rounds of seeding. Crystals of native PatF were grown under the same conditions, but were of poorer diffraction quality.

A single crystal was cryoprotected in a solution of mother liquor supplemented with 30% glycerol and was flash-cooled in a stream of nitrogen at 100 K. A single-wavelength anomalous dispersion (SAD) data set was collected at the Se *K* absorption edge (0.979 Å wavelength) at 100 K on beamline I04 at DLS. The data were processed and scaled in *xia*2 (Winter, 2010 ▶) using *XDS* (Kabsch, 2010 ▶) and *SCALA* (Evans, 2006 ▶) to a resolution of 2.13 Å. The structure was solved using *AutoSol* in *PHENIX* and automated model building of the chains was carried out using *AutoBuild* in *PHENIX* (Adams *et al.*, 2010 ▶). The model was refined by iterative cycles of manual rebuilding using *Coot* (Emsley *et al.*, 2010 ▶) and refinement using *REFMAC*5 (Murshudov *et al.*, 2011 ▶) in the *CCP*4 suite (Winn *et al.*, 2011 ▶). TLS restraints were calculated using the *TLSMD* server (Painter & Merritt, 2006 ▶) and were used in refinement (Winn *et al.*, 2001 ▶). *PISA* was used to assess the oligomeric state of the protein (Krissinel & Henrick, 2007 ▶). The structure was validated using *MolProbity* (Chen *et al.*, 2010 ▶) and the coordinates were deposited in the Protein Data Bank (PDB entry 4bg2).

Homology models of LynF and TruF1 were created using the ‘one 2 one threading’ module of *Phyre*2 (Kelley & Sternberg, 2009 ▶) by inputting the sequence of interest and the SeMet PatF structural coordinates.

Structure alignments were carried out using the SSM Superpose feature of *Coot* and all structures were presented using *PyMOL* (DeLano, 2002 ▶), with the exception of the electrostatic potential maps, which were presented using *CCP*4*mg* (McNicholas *et al.*, 2011 ▶).

Sequence alignments were carried out using *ClustalW* (Larkin *et al.*, 2007 ▶) and were presented using *ALINE* (Bond & Schüttelkopf, 2009 ▶).

### 2.3. Biological assays

Assays were set up to assess potential prenylation by PatF under conditions adapted from McIntosh *et al.* (2011 ▶). PatF at a concentration of 4–10 µ*M* was incubated with 0.1–1 m*M* Boc amino-acid derivative (Boc-Ser, Boc-Tyr or Boc-Trp), 10 m*M* HEPES pH 7.5, 10–­100 m*M* MgCl_2_, 3 m*M* tris(2-carboxyethyl)phosphine (TCEP) and 0.5–1 m*M* DMAPP. All reactions were incubated at 310 K for either 24 h or 7 d. Reactions were analysed by MS.

## 3. Results

### 3.1. Overall protein structure

**Figure 2:**
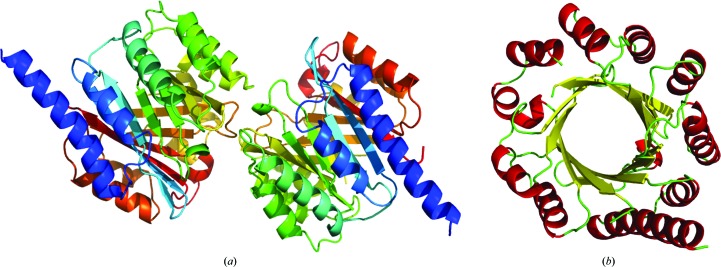
X-ray crystal structure of PatF. (*a*) ‘Chainbows’ representation of the asymmetric unit showing the presence of two molecules. (*b*) Secondary-structure representation of the PatF monomer; α-helices are shown in red and β-sheets are shown in yellow. PatF is formed by a 12-stranded antiparallel β-barrel surrounded on the outside by 12 α-helices, with α-helix 1 protruding out from the rest of the structure.

The protein crystals belonged to space group *P*2_1_, with two biological monomers in the asymmetric unit (Fig. 2 ▶ *a*). Four Se sites, corresponding to two selenomethionines per monomer, were identified. The third selenomethionine residue in each monomer is positioned in a disordered loop.

PatF is formed by a 12-stranded antiparallel β-barrel surrounded on the outside by 12 α-helices in a similar manner to the TIM-barrel motif (Banner *et al.*, 1976 ▶; Fig. 2 ▶ *b*); α-helix 1 protrudes out from the remainder of the structure. There is a single disulfide bridge at the C-­terminus linking Cys276 and Cys307. The final refined model contains residues 3–196 and 205–307 of chain *A* and residues 2–196 and 205–307 of chain *B*. The missing residues are found on the connecting loop between β8 and α8 and also at the N- and C-termini, all of which are presumed to be disordered. PatF is a globular protein with approximate dimensions of 45 × 43 × 53 Å. Analysis with *PISA* suggests that PatF exists as a monomer in solution, consistent with gel filtration. Full data-collection and refinement statistics can be found in Table 1 ▶.

**Figure 3:**
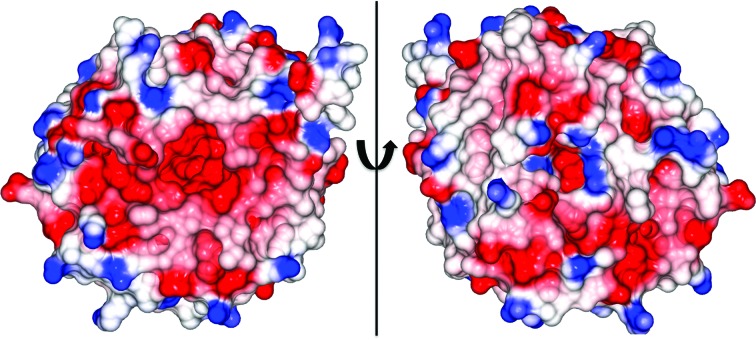
Electrostatic surface-potential map of PatF rotated around 180°. The central pore of the β-barrel, which is the presumed binding site, is highly electronegative (red), with only minor electropositive patches (blue) found at the pore entry.

Electrostatic surface-potential representations of PatF highlight a highly electronegative central pore that could disfavour binding of the electronegative DMAPP (Fig. 3 ▶). Attempts to solve a crystal structure of a complex with DMAPP were unsuccessful, with no electron density for the ligand present in any of the structures.

### 3.2. Biological assays

Prenylation assays were set up using PatF with the Boc amino-acid derivatives Boc-Ser, Boc-Tyr and Boc-Trp. In all reactions only the original starting masses of both the Boc amino acid and the DMAPP molecule were observed by MS, confirming a lack of prenylation.

### 3.3. Homology-model building

**Figure 4:**
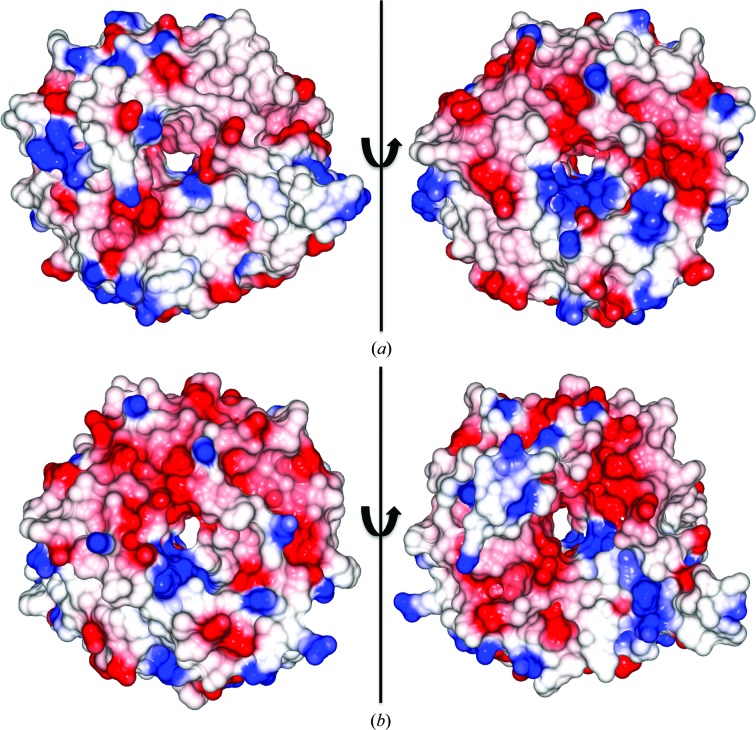
Electrostatic surface-potential maps of two PatF homologues. The structures of these proteins were generated using ‘one 2 one threading’ in *Phyre*2. Maps are rotated 180° to view both sides of the pore. (*a*) LynF, (*b*) TruF1. In contrast to PatF, both enzymes have electropositive patches in the central pore of the β-barrel.

To further clarify potential DMAPP binding, we sought to generate homology models of related cyanobactin family members which have been confirmed to function as prenyltransferases. LynF and TruF1 homology models have electrostatic surface potential maps with clear electropositive regions. Neither has the same highly electronegative character as PatF (Fig. 4 ▶).

### 3.4. Structure overlays and sequence alignments

**Figure 5:**
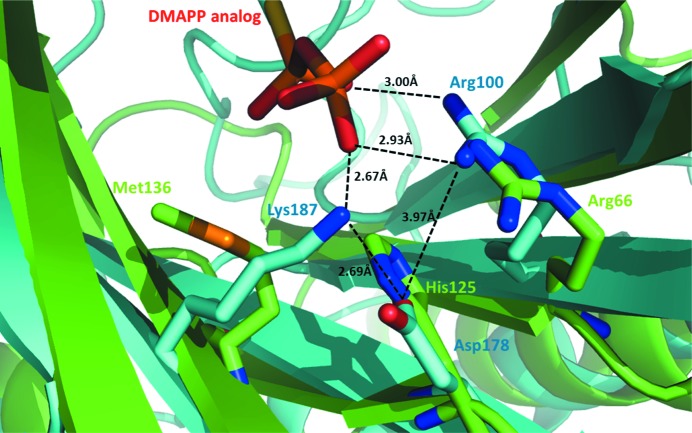
Structural alignment of PatF (green) and DMATS (cyan) highlighting the key residues involved in DMATS–DMAPP binding and their absence in PatF. Lys187 of DMATS forms a salt bridge to the phosphate O atom of the DMAPP mimic (2.67 Å), while Asp178 forms a stabilizing interaction with Arg100, which in turn forms a salt bridge to the DMAPP mimic. In PatF, Lys187 and Asp178 correspond to Met136 and His125, respectively. These residue changes would abolish these DMAPP-binding interactions.

To further characterize the potential binding site of PatF, we performed structural alignments with dimethylallyl tryptophan synthase (DMATS) from *Aspergillus fumigatus* (Metzger *et al.*, 2009 ▶; PDB entry 3i4x). DMATS is a known prenyltransferase and its structure was solved in the presence of both the amino acid and a DMAPP mimic. DMATS is considerably different from PatF in sequence homology (<5%), yet both contain the same characteristic β-barrel pore surrounded by α-helices. Focusing on the DMAPP-binding site allowed us to assess which interactions may be involved in DMAPP binding in PatF and indeed in related cyanobactin family members (Fig. 5 ▶). Two key interactions of DMATS were identified to involve Lys187 and Asp178, which both play a role in binding DMAPP. Lys187 forms a strong salt bridge to the phosphate O atom (2.6 Å), while Asp178 forms a stabilizing interaction with Lys187 and also with Arg100, which in turn forms a salt bridge to the DMAPP molecule. In PatF, the structurally equivalent residues to Lys187 and Asp178 are Met136 and His125, respectively. These significant changes mean that Met136 would not form the same salt-bridge interaction as the lysine in DMATS, while His125 would have a repulsive effect on Arg66 (the equivalent of Arg100 in DMATS) rather than a stabilizing interaction.

**Figure 6:**
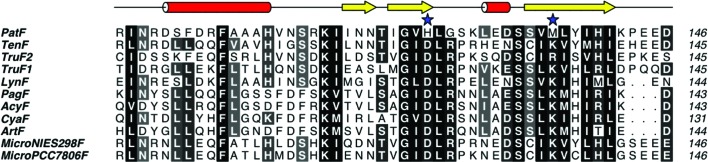
Partial *ClustalW* sequence alignment of PatF and related cyanobactin family members. The key residue changes in PatF, Met136 and His125, are marked by stars. The secondary-structure elements of PatF are displayed.

Upon examination of sequence alignments of the PatF family of enzymes from cyanobactin pathways, Met136 and His125 of PatF are found to correspond to Lys/Arg and Asp, respectively (*i.e.* they are closely related to those in DMATS; Fig. 6 ▶; a full sequence alignment is available in the Supplementary Material^1^). As LynF and TruF1 are active using DMAPP as a substrate, we suggest that the amino-acid substitutions in PatF lead to its inability to bind DMAPP and lack of prenyltransferase activity. Gly127 in PatF corresponds to an arginine residue in the other cyanobactin family members and the structurally equivalent residue in DMATS is a lysine (Lys180). Lys180 in DMATS and Gly127 in PatF are both sited at the pore opening in their respective structures but do not appear to be directly involved in binding. Attempts to mutate PatF in order to design in an active site based on the other cyanobactin proteins resulted in insoluble protein expression only. The single mutant M136K and the triple mutant H125D/G127R/M136K were insoluble when expressed under the same conditions as native PatF.

## 4. Discussion

The crystal structure of PatF has been solved and comprises a β-­barrel core surrounded by α-helices, which is a known motif for prenyltransferases. We have created electrostatic potential maps, performed structural alignments with a prenyltransferase in complex with DMAPP, examined sequence alignments with related proteins and assessed prenylation in biological assays. Our data, together with the lack of prenylation of any natural patellamide, indicate that PatF is an inactive prenyltransferase.

Donia *et al.* (2006 ▶) have reported that PatF is essential for patellamide production *in vivo* and therefore it must be responsible for another function. As the structure does not give any insight into what role this may be, further study will be required.

## Supplementary Material

PDB reference: PatF, 4bg2

Full sequence alignment of PatF and related cyanobactin family members.. DOI: 10.1107/S1744309113012931/gx5216sup1.tif

## Figures and Tables

**Table 1:** Data-collection and refinement statistics for SeMet PatF (PDB entry 4bg2) Values in parentheses are for the highest resolution shell (where applicable).

Data collection	
Beamline	I04, DLS
Wavelength (Å)	0.979
Space group	*P*2_1_
Unit-cell parameters (Å, °)	*a* = 47.08, *b* = 135.80, *c* = 48.74, α = γ = 90.0, β = 118.5
Resolution range (Å)	35.32–2.13 (2.19–2.13)
Total No. of reflections	280235
No. of unique reflections	29355
Multiplicity	9.5 (9.8)
Completeness (%)	97.9 (97.5)
Mean *I*/σ(*I*)	14.0 (3.5)
*R*_merge_	0.10 (0.69)
Anomalous completeness (%)	97.9 (97.6)
Anomalous multiplicity	4.8 (4.9)
Refinement	
*R*_work_/*R*_free_ (%)	19.0/22.1
R.m.s.d., bond lengths (Å)	0.009
R.m.s.d., angles (°)	1.314
No. of atoms	
Protein	4865
Water	86
*B* factors (Å^2^)	
Protein	41.6
Water	40.0

## Footnotes

1 Supplementary material has been deposited in the IUCr electronic archive (Reference: GX5126).
