# Supplementary figures and images for: Structure of PatF from Prochloron didemni
Source: Acta Crystallogr Sect F Struct Biol Cryst Commun. 2013 May 23;69(Pt 6):618–23. doi: 10.1107/S1744309113012931 (PMC3668578; doi:10.1107/S1744309113012931)

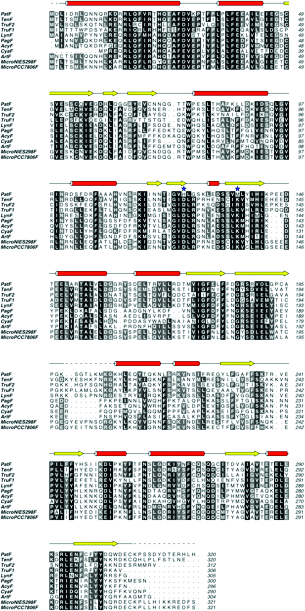

Supplement: Supplementary file 1 [file f-69-00618-sup1.tif]
